# Prevalence of SARS-CoV-2 infection in Italian pediatric population: a regional seroepidemiological study

**DOI:** 10.1186/s13052-021-01074-9

**Published:** 2021-06-05

**Authors:** Manola Comar, Simone Benvenuto, Marzia Lazzerini, Giorgio Fedele, Egidio Barbi, Alessandro Amaddeo, Francesco Maria Risso, Tamara Strajn, Paola Di Rocco, Paola Stefanelli, Giovanni Rezza

**Affiliations:** 1 Institute for Maternal and Child Health, IRCCS “Burlo Garofolo”, Trieste, Italy; 2 University of Trieste, Trieste, Italy; 3 Italian National Institute of Health, Rome, Italy

**Keywords:** Seroprevalence, SARS-CoV-2, IgG antibodies, Pediatric

## Abstract

**Background:**

Data on the effective burden of the SARS-CoV-2 pandemic in pediatric population are very limited, mostly because of the higher rate of asymptomatic or paucisymptomatic cases among children. Updated data on COVID-19 prevalence are needed for their relevance in public health and for infection control policies. In this single-centre cross-sectional study we aimed to assess prevalence of SARS-CoV-2 infection through IgG antibodies detection in an Italian pediatric cohort.

**Methods:**

The study was conducted in January 2021 among both inpatients and outpatients referring to Research Institute for Maternal and Child Health “Burlo Garofolo” in Trieste, Friuli Venezia-Giulia, Italy, who needed for blood test for any reason. Collected samples were sent to Italian National Institute of Health for analysis through chemiluminescent immunoassay (CLIA).

**Results:**

One hundred sixty-nine patients were included in the study, with a median age of 10.5 ± 4.1 years, an equal distribution for sex (49.7% female patients), and a 55.6% prevalence of comorbidities. Prevalence of anti-SARS-CoV-2 trimeric Spike protein IgG antibodies was 9.5% (*n* = 16), with a medium titre of 482.3 ± 387.1 BAU/mL. Having an infected cohabitant strongly correlated with IgG positivity (OR 23.83, 95% CI 7.19–78.98, *p* < 0.0001), while a cohabitant healthcare worker wasn’t associated with a higher risk (OR 1.53, 95% CI 0.4–5.86, p 0.46). All of the 5 patients who had previously tested positive to a nasopharyngeal swab belonged to the IgG positive group, with a 3-month interval from the infection at most.

**Conclusion:**

We assessed a 9.5% SARS-CoV-2 seroprevalence in a pediatric cohort from Friuli Venezia-Giulia region in January 2021, showing a substantial increase after the second peak of the pandemic occurred starting from October 2020, compared to 1% prevalence observed by National Institute of Statistics (ISTAT) in July 2020.

## Background

At the end of December 2019, a severe acute respiratory syndrome caused by a coronavirus 2 (SARS-CoV-2) emerged in Wuhan (Hubei, China) [1], rapidly spreading worldwide thereafter. On March 11th 2020 WHO assessed the outbreak as a pandemic. Italy was the first European country to be hit by the pandemic [2], being for weeks the second in the world, after China, for number of identified cases. As of March 2021, 3 million cases have been confirmed in Italy, with about 100.000 deaths (https://www.epicentro.iss.it/coronavirus/sars-cov-2-sorveglianza-dati).

Despite the high number of infected people, data on the effective burden of the pandemic in pediatric population are very limited, mostly because of the higher rate of asymptomatic or paucisymptomatic cases among children. Given the rapid evolution of the pandemic during winter season, updated data on pediatric COVID-19 prevalence are needed for their relevance in public health and in particular for infection control policies.

Although its role in COVID-19 diagnosis is still poorly defined, especially among children, detection of anti-SARS-CoV-2 IgG antibodies represents a useful method to assess epidemiological information in the general population. The aim of this single-centre cross-sectional study was to assess prevalence of SARS-CoV-2 infection through IgG antibodies detection in an Italian pediatric cohort.

## Methods

The study was conducted in January 2021 among children who accessed the Research Institute for Maternal and Child Health “Burlo Garofolo” in Trieste, Friuli Venezia-Giulia, Italy. Both inpatient and outpatients referring to pediatric wards, day hospital, and emergency department were included, based on two inclusion criteria: age < 18 years and need for blood test for any reason. Refusal to participate to the study, risk factors for ongoing SARS-CoV-2 infection (typical symptoms, recent positive nasopharyngeal swab, contact with confirmed or suspected cases, travelling abroad), and/or immunodeficiency, either primitive or secondary, were considered as exclusion criteria. Drug-induced immune suppression was defined for patient receiving chemotherapy, rituximab, immune globulins, and/or glucocorticoids such as methylprednisolone 1 mg/Kg or equivalent for at least 14 days. Medical staff obtained informed consent from parents, who were asked to fill an anamnestic form with relevant clinical and epidemiological data regarding their children. Specific test tubes were collected at the same time of the planned blood samples, then anonymized and sent to the Italian National Institute of Health for analysis. Anti-SARS-CoV-2 trimeric Spike protein IgG antibodies detection was performed through chemiluminescent immunoassay (CLIA) (Diasorin, Italy). Results are expressed as absolute values and percentage; *p* values were assessed using Fisher’s test.

## Results

**Fig. 1:**
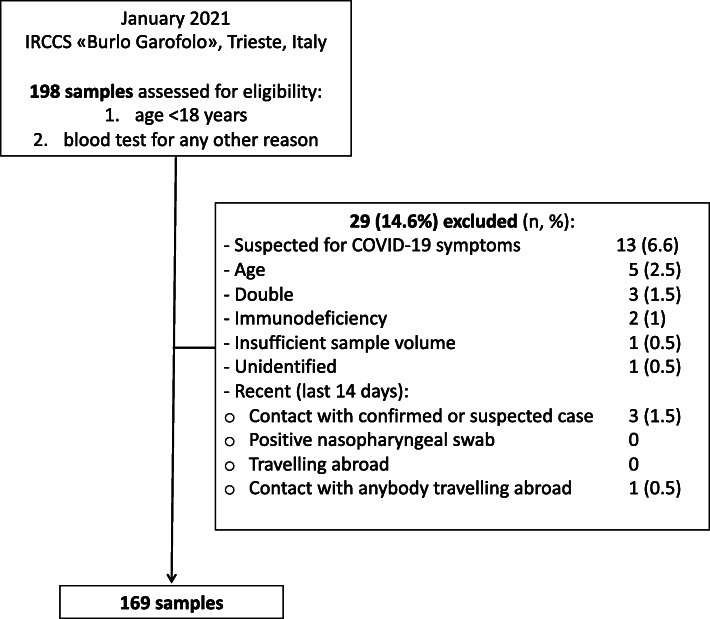
Flow chart of study population

Between January 5th and 31st 2021, a total number of 198 samples was collected. Among these, 29 samples were excluded, mostly because of the simultaneous presence of suspected for COVID-19 symptoms or because age exclusion criterion was met (Fig. 1). Characteristics of the 169 remaining patients are shown in Table 1. Median age was 10.5 ± 4.1 years; all age intervals were well represented in the cohort, with children ranging from 12 to 17 years old being the most prevalent (47.3%). An equal distribution for sex was observed, with 84 (49.7%) female patients. Most of the children had Italian citizenship (154, 91%). Approximately half of them presented at least one comorbidity (94, 55.6%), with syndromes and/or cerebral palsy being the most prevalent (22, 23.4%). Children with a cohabitant healthcare worker accounted for 13.4%.

**Table 1:** Characteristics of enrolled patients

Characteristic	No of patients (***n*** = 169) (%)
**Department**	
- Day Hospital	77 (45.6)
- Pediatric Surgery	36 (21.3)
- Pediatrics	24 (14.2)
- Gastroenterology	14 (8.3)
- Blood Drawing Center	12 (7.1)
- Emergency Department	6 (3.5)
**Age**	
- Mean ± SD	10.5 ± 4.1
- ≤ 5	28 (16.6)
- 6–11	61 (36.1)
- 12–17	80 (47.3)
**Sex**	
- M	85 (50.3)
- F	84 (49.7)
**Citizenship**	
- Italy	154 (91)
- Romania	4 (2.4)
- Serbia	4 (2.4)
- Albania	2 (1.2)
- China	1 (0.6)
- Pakistan	1 (0.6)
- Spain	1 (0.6)
- Turkey	1 (0.6)
- UK	1 (0.6)
**Comorbidities**	94 (55.6)
- Malformation/Syndrome/Cerebral Palsy	22 (23.4)
- Obesity	12 (12.8)
- Prematurity	10 (10.6)
- Respiratory	10 (10.6)
- Cardiac	5 (5.3)
- Diabetes	4 (4.3)
- Other	31 (33)
**Cohabitant healthcare worker**	23 (13.6)
**Having an infected cohabitant**	20 (11.8)
- 1 month before	7
- 2 months before	5
- 3 months before	5
- More than 3 months before	1
- Unspecified	2
- Tested by nasopharyngeal swab	20 (100)
- Positive	4 (20)
**Nasopharyngeal swab**	
- Tested	122 (72.2)
- Positive	5 (4.1)
○ 1 month before	2
○ 2 months before	2
○ 3 months before	1
○ More than 3 months before	0
**Anti-SARS-CoV-2 Spike Protein IgG Antibodies**	
- Positive	16 (9.5)
- Negative	153 (90.5)

At least one cohabitant infected with SARS-CoV-2 in the last months was reported by 20 patients (11.8%). All of these children had been tested with a nasopharyngeal swab and 4 (20%) had resulted positive. One more patient, without any infected cohabitant, had previously tested positive at the nasopharyngeal swab, globally reaching a 4.1% prevalence in our cohort.

Among 169 analyzed samples, 16 (9.5%) children tested positive for anti-SARS-CoV-2 trimeric Spike protein IgG antibodies, with a medium titre of 482.3 ± 387.1 BAU/mL. Their characteristics are shown in Table 2. Most of them were outpatients. The median age (10.3 ± 4.1 years) was comparable with the entire cohort. A slightly higher prevalence was observed in girls (56.3%). Having a cohabitant healthcare worker (18.7%) was not associated with a higher risk of antibodies positivity (OR 1.53, 95% CI 0.4–5.86, p 0.46). Remarkably, 10 patients of this subgroup (62.5%) reported an infected cohabitant in the last months, so that a statistically significant correlation was observed (OR 23.83, 95% CI 7.19–78.98, *p* < 0.0001).

**Table 2:** Characteristics of patients testing positive for anti-SARS-CoV-2 Spike protein IgG antibodies

Antibodies titre	BAU/mL
- Mean ± SD	482.3 ± 387.1
- Median	387.4
- Interquartile range	147.3–650.6
- Max	1331.2
- Min	87.9
**Department**	
- Day Hospital	8 (50)
- Pediatric Surgery	4 (25)
- Blood Drawing Center	2 (12.5)
- Pediatrics	1 (6.25)
- Gastroenterology	1 (6.25)
- Emergency department	0
**Age**	
- Mean ± SD	10.3 ± 4.1
- ≤ 5	3 (18.7)
- 6–11	6 (37.6)
- 12–17	7 (43.7)
**Sex**	
- M	7 (43.7)
- F	9 (56.3)
**Citizenship**	
- Italy	15 (93.7)
- Albania	1 (6.4)
**Comorbidities**	12 (75)
- Malformation/Syndrome/Cerebral Palsy	4 (33.4)
- Obesity	3 (25)
- Prematurity	2 (16.7)
- Respiratory	1 (8.3)
- Diabetes	1 (8.3)
- Cardiac	0
- Other	1 (8.3)
**Cohabitant healthcare worker**	3 (18.7)
- OR, 95% CI, p value	1.53, 0.4–5.86, 0.46
**Having an infected cohabitant**	10 (62.5)
- OR, 95% CI, *p* value	23.83, 7.19–78.98, < 0.0001
- 1 month before	6 (60)
- 2 months before	1 (10)
- 3 months before	3 (30)
- More than 3 months before	0
- Tested by nasopharyngeal swab	10 (100)
- Positive	4 (40)
**Nasopharyngeal swab**	
- Tested	15 (93.7)
- Positive	5 (30)
○ 1 month before	2 (40)
○ 2 months before	2 (40)
○ 3 months before	1 (20)
○ More than 3 months before	0

Most of the IgG positive patients had received at least one nasopharyngeal swab (15, 93.7%). All of the 5 children who had previously tested positive at the swab in our cohort belonged to the IgG positive group, accounting for 30%.

## Discussion

To date, this is the first antibody epidemiological study to investigate SARS-CoV-2 infection prevalence in Italian pediatric population after the second peak of the epidemic occurred starting from October 2020.

Data from the Italian case-based surveillance system showed that at the end of the first peak in May 2020 pediatric patients accounted for 1.8% of total confirmed COVID-19 infections, in a population where 16% is < 18 years of age [3]. These data were in accordance with the probable inferior susceptibility to SARS-CoV-2 infection (estimated as approximately half compared to adults) and the higher prevalence of paucisymptomatic (around 80% in the 10–19 years group) among pediatric patients [4]. As a matter of fact, both reduced susceptibility and reduced symptoms represent two major obstacles in defining pediatric infection prevalence. This underlines the fundamental role of serology in understanding the real burden of the pandemic. Various antibody tests have been validated so far, reaching their highest sensitivity for detecting previous SARS-CoV-2 infection when used at least 15 days after the onset of potential symptoms [5]. Reported positive percent agreement (PPA) of our test, in particular, was 98.7% (95% CI 94.5–99.6%).

A few studies have evaluated SARS-CoV-2 seroprevalence in adult Italian cohorts. As an example, Vena et al. [6] estimated a medium 11% IgG and/or IgM positivity in a large population from five administrative departments of Liguria and Lombardia regions, which were among the hardest hit by the pandemic, in April 2020. Citizen above 10 years of age from the Autonomous Province of Trento, which carried the highest incidence of COVID-19 cases, were studied in May 2020 with a resulting IgG antibodies prevalence of 23.1% [7]. Finally, between May and July 2020, the Italian National Institute of Statistics (ISTAT) assessed an overall 2.5% seroprevalence in a large sample from 2000 Italian municipalities, with data from Friuli Venezia-Giulia region showing a 1% prevalence (http://www.salute.gov.it/imgs/C_17_notizie_4998_0_file.pdf). No recent studies have assessed antibody prevalence evolution in the general Italian population, and in the Friuli Venezia-Giulia in particular.

Concerning this region, a reasonable but large esteem of the burden of the infection could be based on latest mortality data, that is almost 3000 deaths so far, which on a medium lethality rate around 1% [8] indicate approximately 300 thousand infected people over 1.2 million residents so far, with an expected 30% seroprevalence. As uncertain as this esteem could be, our study reports an at least 3-folds inferior seroprevalence in pediatric population.

Our cohort indirectly demonstrates the low sensitivity of our current surveillance system: among 15 previously infected children receiving a swab at some point in their past, only 30% had actually been found positive. However, samples from all of the 5 children who had tested positive to a nasopharyngeal swab were actually found positive for SARS-CoV-2 IgG antibodies, with a 3-month interval from the infection at most in our cohort. This is in accordance with previous observations in adult cohorts, where SARS-CoV-2 antigen-specific antibodies have been found in 90% of patients up to 8 months after infection, although not predictive of T cell memory and with substantial heterogeneity among individuals [9].

As expected, having an infected cohabitant strongly correlates with IgG positivity, confirming an intrafamilial model of transmission, which is helpful in detecting asymptomatic cases or suspecting late manifestations of the infection such as multisystem inflammatory syndrome in children (MIS-C) or the hypothesized persistence of COVID-19 symptoms, the so-called “Long COVID” [10]. Moreover, our observation that 10/20 (50%) children reporting an infected household resulted IgG positive is in accordance with previous studies on the topic [11].

No correlation was found between IgG positivity and having a cohabitant healthcare worker, suggesting that the expected greater exposure to contagion is not currently reflecting a higher risk of infection.

The two major limitations of this study are that our sample may suffer from a selection bias and not fully represent the general pediatric population, owing to a considerable prevalence of comorbidities, and the limited dimension of our sample.

## Conclusions

In a single-centre prospective study, we assessed a 9.5% prevalence of anti-SARS-CoV-2 Spike protein IgG antibodies suggesting that children are actually considerably less infected than adults. This result is significantly higher than observed seroprevalence after the first peak of the pandemic in July 2020 by National Institute of Statistic (ISTAT). IgG antibodies were found at a maximum 3-months interval from the infection. Having an infected cohabitant is strongly associated with IgG antibodies detection, while cohabitant healthcare workers don’t seem to carry a greater risk. These data could be helpful to plan better infection control policies also considering the possible impact of SARS-CoV-2 variant on this group.

## Data Availability

The datasets generated and/or analyzed during the current study are not publicly available due to individual privacy reasons.

## Abbreviations

SARS-CoV-2: Severe Acute Respiratory Syndrome Coronavirus 2
WHO: World Health Organization
COVID-19: Coronavirus Disease 2019
CLIA: Chemiluminescent Immunoassay
OR: Odds Ratio
CI: Confidence Interval
PPA: Positive Percent agreement
ISTAT: National Institute of Statistics
